# Lemierre's Syndrome: A Case Report

**DOI:** 10.7759/cureus.14713

**Published:** 2021-04-27

**Authors:** Hannah Doan, Sara Niyazi, April Burton, Ikramamul Nibir, Augustas Kavaliauskas

**Affiliations:** 1 Medicine, Lake Erie College of Osteopathic Medicine, Bradenton, USA; 2 Internal Medicine, AdventHealth Florida, Sebring, USA

**Keywords:** lemierre's syndrome, septic thrombophlebitis, necrobacillosis, jugular vein suppurative thrombophlebitis, post-anginal sepsis, postanginal sepsis

## Abstract

Lemierre’s syndrome is a condition in which an oropharyngeal infection progresses to sepsis and thrombophlebitis of the internal jugular vein. Although the incidence of this syndrome has fallen dramatically since the widespread use of antibiotic therapy to treat streptococcal pharyngitis, it should still be suspected in otherwise healthy young patients presenting with the triad of prolonged pharyngitis, lateral neck pain, and septic symptoms. In this report, we explore a unique case of Lemierre’s syndrome complicated by hypercoagulability and ineffective initial antibiotic therapy.

## Introduction

Lemierre’s syndrome, also known as jugular vein suppurative thrombophlebitis, post-anginal sepsis, and necrobacillosis, was first described by Andre Lemierre in 1936 and is characterized by thrombophlebitis of the internal jugular vein with concurrent sepsis [1,2]. The highest rates of Lemierre's syndrome were seen during the pre-antibiotic era, but since the use of antibiotics to treat oropharyngeal infections, it has become a fairly rare occurrence, with recent studies estimating a rate of approximately 3.6 cases per 1 million individuals per year [3]. It is most commonly preceded by pharyngitis, but other reported events leading to Lemierre’s syndrome include dental infections, tonsillitis, infectious mononucleosis, and intravenous catheter insertion [4]. Infection usually descends from its primary site in the oropharynx to the carotid sheath vessels in the lateral pharyngeal space, causing septic thrombophlebitis of the internal jugular vein and sometimes the internal carotid artery [3]. As the infection progresses, it commonly spreads from the internal jugular vein to the lung, causing cavitary lung abscesses or empyema [5]. There have also been reports of spread to the joints, liver, muscle, pericardium, brain, and skin in some cases [6].

Lemierre’s syndrome is usually a polymicrobial infection, with the most commonly implicated organisms being *Fusobacterium necrophorum*, *Eikenella corrodens*, *Porphyromonas asaccharolytica*, *Streptococcus pyogenes*, and *Bacteroides* [7-9]. Treatment requires appropriate antibiotic therapy combined with surgical intervention and anticoagulation when indicated [10]. It is recommended that empiric therapy include a beta-lactamase resistant beta-lactam antibiotic, such as ampicillin-sulbactam, piperacillin-tazobactam, or meropenem [11]. In rare instances, surgical ligation or excision of the internal jugular vein may be warranted [12].

## Case presentation

A previously healthy 21-year-old Caucasian female presented to the emergency department in a hypotensive and febrile state with associated weakness and vomiting. She reported having a sore throat and fever three days prior, for which she went to urgent care and was prescribed azithromycin for bilateral tonsillitis. Over the course of three days, the azithromycin did not relieve her symptoms, and instead, they progressed to also include nausea and vomiting. On the third day, she described a sensation of feeling “something burst” in her neck, which improved her sore throat but lead to the presence of blood in her sputum, prompting her visit to the emergency department.

**Figure 1:**
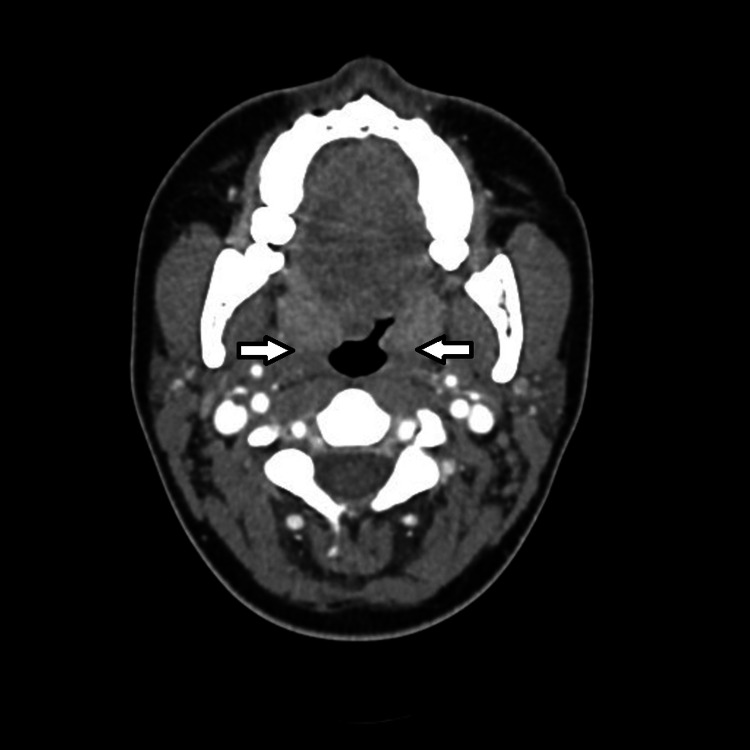
Initial CT with contrast demonstrating bilateral palatine tonsil enlargement.

**Figure 2:**
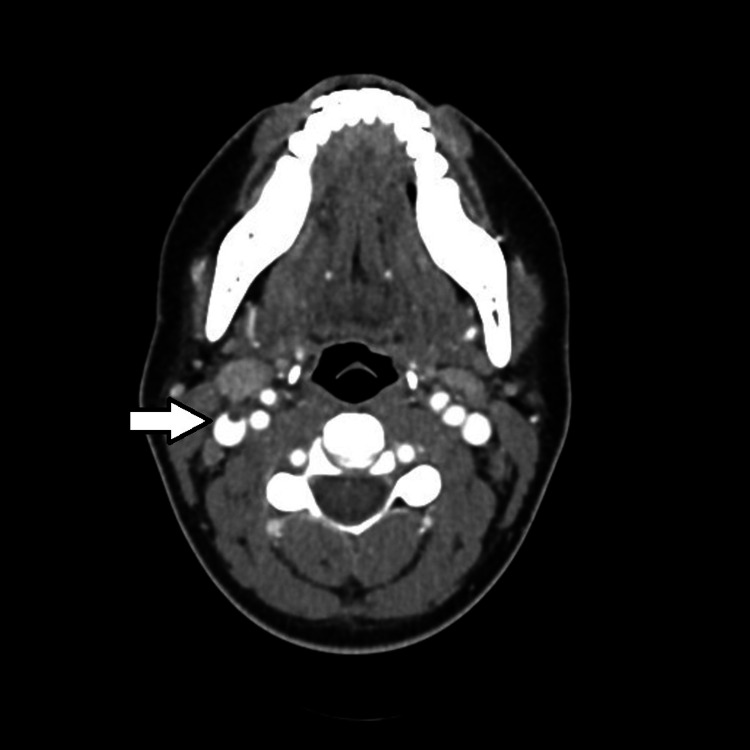
Initial CT with contrast demonstrating a filling defect in the right internal jugular vein.

On initial assessment, she had a fever of 104^o^F, a blood pressure of 79/39 mmHg, a heart rate of 134 bpm, and a swollen and tender right neck. Her laboratory evaluation showed leukopenia and thrombocytopenia, and a contrast CT scan of the neck revealed enlarged bilateral palatine tonsils and a filling defect in the right internal jugular vein (Figures 1-2). The combination of her physical exam, laboratory, and image findings pointed to the diagnosis of Lemierre’s syndrome with progression to sepsis. She was promptly admitted to the critical care unit and started on vancomycin and piperacillin/tazobactam.

**Figure 3:**
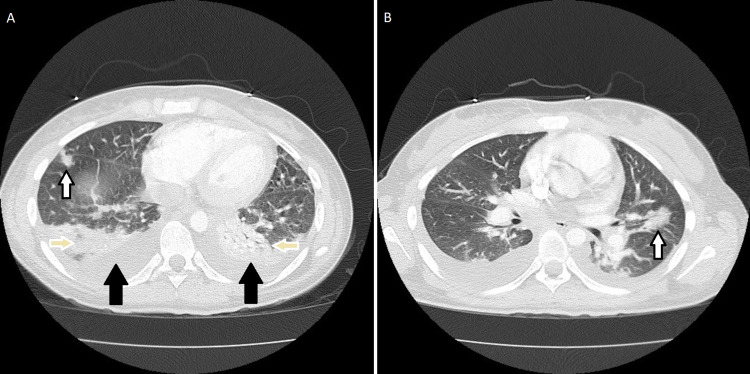
Chest CT with contrast demonstrating pleural effusion (black arrows), consolidation (yellow arrows) and nodular opacities (white arrows).

On the second day of admission, her fever persisted and she developed acute hypoxic respiratory failure. A chest CT with contrast was performed and showed bilateral pleural effusion, patchy consolidations, and nodular opacities suggestive of lung abscess (Figure 3). She was then started on furosemide and IV meropenem to treat her pleural effusions and abscesses, respectively. Her fever, sore throat, and inflammation resolved in response to the antibiotics, however, her thrombus in the right internal jugular vein persisted. She was in a continual state of hypercoagulability, so the decision was made to have her Nexplanon® etonogestrel implant removed. Over the next two days, her condition started to improve with a decrease in her C-reactive protein (CRP) and an increase in her platelet count and blood pressure. At this point her throat pain, fever, and vomiting had resolved, but her right neck and ear were tender to palpation and she complained of mild chest tightness.

**Figure 4:**
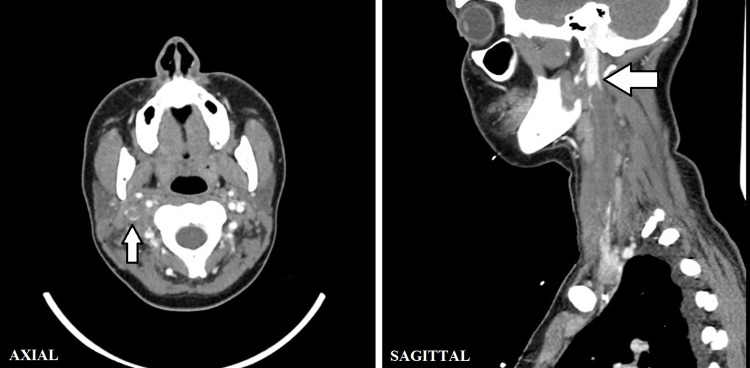
Axial and sagittal view of CT with contrast demonstrating a fully occluded right internal jugular vein (white arrows).

Unfortunately, her condition declined once again the following day, with recurrent fever and worsening neck pain. Repeat CT showed progression of the right internal jugular vein thrombosis into a fully occluded vessel, with wall thickening and hyperemia indicating thrombophlebitis (Figure 4). The thrombophlebitis extended inferiorly to the infrahyoid muscle and supraclavicular fat, and her temperature continued to increase from 100.7^o^F to 101.1^o^F. While the thrombophlebitis was extensive, there were no signs of neck abscess or acute odontogenic infection. At this point, heparin was started as her thrombocytopenia had improved since intake.

In an effort to prevent septic embolism, vascular surgery was consulted and a surgical ligation was planned. However, during the ligation procedure, the patient was found to have severe inflammation and purulent material within the right internal jugular vein, so drainage and a suppurative thrombectomy were performed instead of the initially planned ligation. The thrombus and purulence from the internal jugular vein were retrieved and sent for culture and pathology, which showed signs of extensive acute inflammation but no organism growth. The thrombectomy was successful, and over the next five days, the patient’s condition greatly improved. She was discharged home with clopidogrel, apixaban, IV meropenem, and vancomycin, which were found to be the optimal antibiotic choices based on sensitivity testing.

## Discussion

Due to the low incidence of Lemierre’s syndrome, it is not commonly seen and therefore may not be considered as a complication when treating patients for oropharyngeal infections. This can potentially lead to inadequate treatment regimens, missed diagnosis, and increased disease complications, as seen in our patient. When she arrived at urgent care she was treated with azithromycin, most likely because they did not suspect complications that could potentially result in Lemierre’s syndrome. However, when a patient presents with symptoms of tonsillitis or other oropharyngeal infections, an alternative treatment to consider is penicillin, as it has been shown to provide stronger coverage against certain oropharyngeal microbes, such as *Fusobacterium necrophorum* [13].

Furthermore, a well-known risk of various birth control methods is the possibility of venous thromboembolic events and coagulopathies [14]. Isolated cases of thrombotic thrombocytopenia have also been reported in patients with progestin contraceptive implants [15]. Our patient had an indwelling progestin implant when she developed Lemierre’s syndrome, which may have contributed to her rapid decline and disruption of hemostasis. On the day of implant removal, her platelet count was 49 x 10^3 ^mL, and following removal, her platelet count began to steadily increase and normalize to 257 x 10^3^ mL four days following removal. These findings suggest a possible connection between her progestin implant and her hematologic changes. 

## Conclusions

Hormonal contraceptives may cause changes in hemostasis and put patients at an increased risk for thromboembolic complications. It is possible that the use of a hormonal birth control implant increased our patient's risk of thrombus formation and played a role in her disease severity. This is evidenced by the fact that, in conjunction with the use of antibiotics, removal of the birth control implant appeared to aid in hemostasis. Furthermore, this case brings attention to the urgency of initial antibiotic therapy for oropharyngeal infections and highlights the importance of choosing a regimen that provides adequate coverage for the multitude of oropharyngeal bacteria, including those with increased resistance to more commonly used antibiotics.
